# Simvastatin, Its Antimicrobial Activity and Its Prevention of Alzheimer’s Disease

**DOI:** 10.3390/microorganisms12061133

**Published:** 2024-06-01

**Authors:** Sudip Dhakal, Ian G. Macreadie

**Affiliations:** 1 Health and Biosecurity, Commonwealth Scientific and Industrial Research Organization (CSIRO), Geelong, VIC 3220, Australia; sudip.dhakal@csiro.au; 2 School of Science, RMIT University, Bundoora, VIC 3063, Australia

**Keywords:** simvastatin, antimicrobial, Alzheimer’s disease

## Abstract

Simvastatin, a blockbuster drug for treating hypercholesterolemia, has multifactorial benefits as an antimicrobial agent and plays a preventative role in reducing the incidence of Alzheimer’s Disease (AD). Although most of the beneficial effects of simvastatin have been attributed to its ability to reduce cholesterol levels, recent scientific studies have suggested that its benefits are largely due to its pleiotropic effects in targeting other pathways, e.g., by inhibiting protein lipidation. There are certain pleiotropic effects that can be predicted from the inhibition of the mevalonate pathway; however, some of the effects of simvastatin in proteostasis lead to reduced levels of amyloid beta, the key contributor to AD. This review discusses the use of simvastatin as an antimicrobial agent and anti-AD drug.

## 1. Introduction

**Figure 1:**
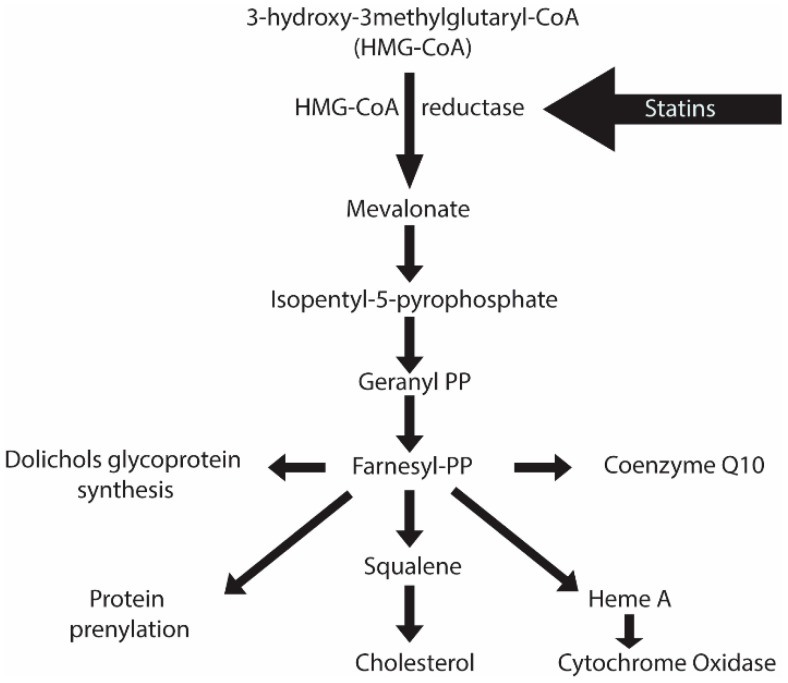
Biosynthetic pathway for cholesterol production. Statins inhibit HMGCR (which reduces cholesterol production) as well as four other important products or processes.

The very high incidence of hypercholesterolemia and its links to heart disease have led to searches for treatments to lower cholesterol production [1]. Since endogenous cholesterol production contributes to most of the body’s cholesterol levels, and with age, our cholesterol levels increase, it is obvious that some of the greatest gains in heart disease prevention can be made with the administration of cholesterol lowering drugs. Great progress has been made through the administration of statins, with the gains coming from the discovery of lovastatin. For a detailed description of the early history of statins, please refer to Akiro Endo’s review on the history of the discovery of statins [2]. In brief, with the knowledge of the involvement of 3-hydroxy-3-methylglutaryl-coenzyme A (HMG-CoA) reductase (HMGCR) in a rate limiting step of cholesterol production (see Figure 1), Akiro Endo developed a targeted radioactive enzymatic assay to screen for HMGCR inhibitors in microbial metabolites. This ultimately led to the discovery of lovastatin, a product of *Aspergillus terreus*. Although lovastatin was shown to be active in lowering cholesterol levels in humans, a chemically modified form of it, simvastatin, is a greatly superior product in terms of its pharmaceutical benefits. Merck marketed simvastatin as Zocor™, one of the first drugs to earn more than one billion USD per annum in sales. Many more statins have now been produced by chemical synthesis; however, simvastatin remains available and is now produced in generic forms. 

## 2. Simvastatin’s Primary Mode of Action—Targeting HMG-CoA Reductase

Simvastatin competitively binds to and inhibits HMGCR; however, this inhibition is partial [3]. Cholesterol is essential to the membranes of cells, and high levels of inhibition of cholesterol synthesis would be deleterious, so new users of statins may be checked for rhabdomyolysis. In a previous study, it was reported that statin usage in conjunction with other drugs could potentiate statin-induced rhabdomyolysis [4]. The toxicity of statin has been well known since 2001, when cerivastatin was withdrawn from the market following 52 deaths associated with statin-induced rhabdomyolysis leading to kidney failure [5]. However, as seen in the schematic above depicting the cholesterol biosynthesis pathway, the inhibition of HMGCR not only inhibits cholesterol production but also affects the production of co-enzyme Q10, heme production, and the prenylation of proteins [6]. Its effects on some of these important products may not be life threatening but they can be enough to alter performance, as reported by athletes [7]. Around 80% of athletes who are prescribed statins discontinue their use because they claim it reduces their performance. Weightlifters have claimed that it reduces their ability to increase muscle mass. Frequently, it is suggested that statin users take co-enzyme Q10 to make up for their reduced performance.

## 3. Antimicrobial Effects of Simvastatin

Statins can also target microbes such as yeast and fungi which contain HMGCR and follow a similar biochemical pathway. While yeast and fungi do not produce cholesterol, in its place they produce ergosterol, which is functionally equivalent. Yeast HMGCR is also inhibited by statins, including simvastatin, such that growth can be totally prevented. This inhibition can be overcome, however, by supplementing the growth medium with ergosterol or cholesterol [8]. This demonstrates the functional equivalence of the two sterols in yeast. It also demonstrates the power of microbial models in the study of pharmaceuticals.

In addition, other components of the biosynthetic pathway shown in Figure 1 are conserved in eukaryotic microbes. For example, yeast produces coenzyme Q6 (functionally equivalent to coenzyme Q10), can prenylate and glycosylate proteins and can utilize cytochrome oxidase in the electron transport chain. 

In summary, simvastatin reduces the levels of ergosterol in yeast and other fungi; however, additional effects may be expected due to other pathways that lie downstream of HMGCR, affecting a range of activities, including mitochondrial function.

We have made a comprehensive survey of the reported antimicrobial effects of simvastatin; these are compiled in Table 1. This table shows a range of antimicrobial effects of simvastatin and provides the concentrations at which effects are observed. Notably, it can be seen that many bacteria are included in this list. It therefore seems reasonable to assume that some of these bacteria may be useful models for study of the actions of simvastatin. However, the similarity between yeast and human cells and the huge amount of knowledge regarding yeast genetics and molecular biology make yeast an ideal model for studying the effects of simvastatin.

Some of the effects were found to occur through a range of activities targeting multiple biosynthetic pathways and cellular functions, including biofilm formation. Thangamamani found widespread antibacterial activity of simvastatin against a range of Gram-positive and Gram-negative bacteria, including MRSA [13]. Simvastatin inhibits the expression of bacterial proteins including toxins, chaperone proteins, and ribosomal proteins, and enhances protein degradation, reduces biofilm formation, inactivates drug efflux pumps, and increases the anti-inflammatory response [13]. Interestingly, simvastatin’s ability to inhibit *S. aureus* biofilm formation is much greater than those of pravastatin or atorvastatin [14]. In addition, recent studies showed the antimicrobial activity of simvastatin in terms of inhibiting the growth of skin infection and periodontal pathogens. In some of these cases, simvastatin’s antimicrobial activity in bacteria was reversed by the addition of cholesterol [19]. Although simvastatin shows broad-spectrum antimicrobial activity against a range of pathogens, it is crucial to keep the effective concentration below the toxic dose [20]. In most of the studies listed in Table 1, the simvastatin peak plasma concentration achieved was lower than the MICs, hence limiting its use as an antimicrobial agent. 

Meanwhile, simvastatin, when used in combination with other antimicrobials, has shown synergistic effects in inhibiting growth of microbes. This approach of combining simvastatin with another antimicrobial agent could be a better way to ensure that simvastatin remains in lower concentration in plasma while having a higher impact compared to using individual antimicrobials. Notably, such combination strategies have the potential to inhibit the growth of multidrug resistant organisms. An example of such is the observed inhibition of *Cryptococcus neoformans* growth when simvastatin was combined with amphotericin B [21]. In another study, simvastatin was reported to enhance Ag^2+^ antimicrobial activity against *Enterococcus faecalis* [22].

## 4. Simvastatin Reduces the Incidence of Alzheimer’s Disease

Currently, around 60–80% of dementia-related deaths are caused by Alzheimer’s Disease (AD) [23]. The disease was named after German psychiatrist Alois Alzheimer presented clinical data of a presenile form of the disease in his talk at the 37th meeting of the Society of Southwest German Psychiatrists in Tübingen on 3rd November 1906, followed by a short paper in 1907 [24]. The disease is characterized by several pathological hallmarks that include the formation of amyloid plaques and neurofibrillary tau tangles, neuroinflammation, loss of proteostasis, mitochondrial dysfunction and impairment of mitochondrial turnover, genomic instability, cognitive impairment, and neuronal death [23,25]. 

Simvastatin has proven its value in reducing cholesterol levels and decreasing the incidence of heart disease and stroke [26,27]. However, through its widespread use, its effects on other human conditions have also been studied. The one that is addressed in this review is its effect on AD. In previous studies, simvastatin has been reported to have multifactorial effects on the major pathological hallmarks of AD.

It has become clear from epidemiological studies that there are some beneficial effects of simvastatin that were not predicted and that may not be linked directly to the cholesterol synthesis pathway described above. A powerful example is the 2007 epidemiological study by Wolozin and colleagues, which showed that simvastatin specifically led to a 50% lowering in the incidence of AD in statin users in the US Veteran’s Affairs administration [28]. It is now nearly two decades since this study was conducted and others have re-looked for this effect. The question of how this effect might occur is the subject of the remainder of this review. Cholesterol levels have a very limited effect on AD, and the effect of simvastatin on AD was unlike those of other statins, which exerted insignificant effects in Wolozin’s study. Therefore, it is important to give a wider consideration to the effects of simvastatin and other statins.

Apart from targeting cholesterol production, there are pleiotropic effects of simvastatin, as shown in Figure 1. One of the side impacts of inhibiting cholesterol pathways is the inhibition of protein prenylation. Protein prenylation is the post-translational modifications of proteins containing the CaaX (where C is a cysteine residue, a is an aliphatic amino acid, and X is any amino acid) motif by isoprenoid lipids [29]. The prenylation process starts with the attachment of either a farnesyl (15-carbon) or a geranylgeranyl (20-carbon) isoprenoid lipid to the cysteine residue of the protein. Proteins are then processed by RAS-converting CaaX endopeptidase 1 (RCE1), followed by isoprenylcysteine carboxymethyl transferase (ICMT), to add an appropriate cap to the modified cysteine residue. The roles of these prenylated proteins are crucial and are well conserved, making them pivotal in neuronal function, synaptic plasticity, and other cellular signaling pathways. A major group of the proteins that are prenylated are the small GTPase family proteins, as the post-translational modification of these proteins determines protein–protein and protein–membrane interactions, which significantly impact cell signaling processes. In this section, some such effects of simvastatin and their correlation with AD are detailed.

### 4.1. Anti-Inflammatory Effect

**Figure 2:**
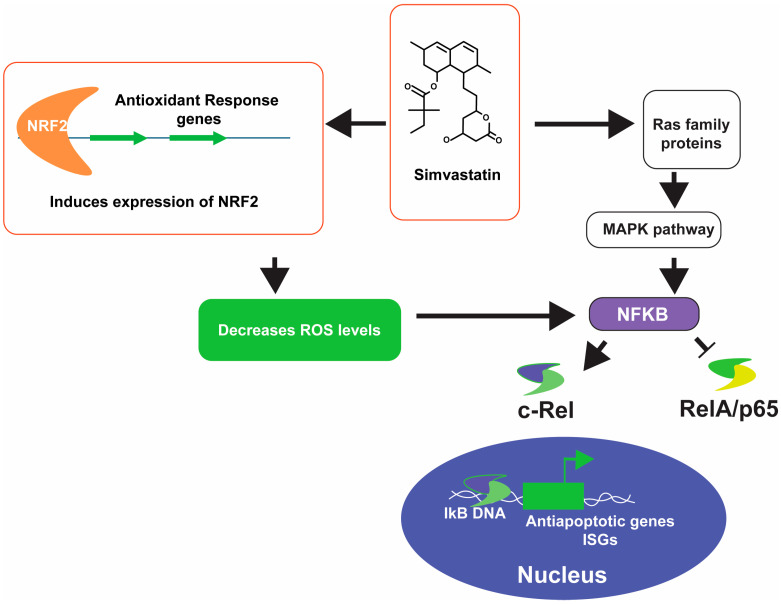
Anti-inflammatory effects of simvastatin and its potential to improve cellular health during AD. Pointed arrows are used to depict the positive impact, and the blunt-arrow is used to show the inhibition of the target. NFκB, Nuclear factor kappa B; NRF2, Nuclear factor erythroid 2-related factor 2; ROS, Reactive oxygen species; MAPK, Mitogen activated protein kinase; IκB, Inhibitor of Kappa B; and ISGs, Interferon stimulating genes.

The important anti-inflammatory effects of simvastatin are summarized in Figure 2. The inhibition of protein prenylation by simvastatin has a direct impact on nuclear factor kappa B (NFκB) activation. In normal conditions, NFκB is bound to inhibitors of NF-κB (IκB) in the cytoplasm and remains inactivated. NFκB regulates adaptive and innate immune responses by taking part in cell survival, activation, and differentiation of immune cells [30]. It is a transcription factor that recognizes IκB DNA elements with consensus sequence 5′-GGGRNWYYCC-3′, where R = A or G, N = A, C, G, or T, W = A or T, and Y = C or T in the promoters/enhancers of target genes which play crucial roles in modulating the cellular immune response [31]. NFκB proteins are proteins of the Rel family including RelA/p65, p52, p50, RelB and c-Rel. Active complexes of NFκB dimers are formed by combinations of these proteins [32]. NFκB has the potential to activate both the protective cellular pathways and the proinflammatory response. The stimulus inside the cell determines the cellular fate via NFκB activation and its interaction with other Rel family proteins. For example, the activation of proinflammatory genes triggers inflammasome formation and activates the inflammatory pathways, while the expression of interferon-stimulating genes (ISGs) has the potential to reprogram the metabolome toward the survival pathway. When a cell receives proinflammatory signals, it has been hypothesized that some IκB gets prenylated, changing its structure and reducing its ability to release and activate NFκB. Activated NFκBs homodimerizes or heterodimerizes and translocates to the nucleus and upregulates the expression of the target genes. In a previous study, it was reported that simvastatin reduced the nucleolar content of the NFκB-RelA complex while increasing NFκB-cRel combination. It is still unclear how simvastatin enriches one combination of the complex in the nucleus while reducing the other. There is a possibility that these protein interactions could have been affected by the inhibition of protein prenylation. However, other researchers have hypothesized that IκB prenylation plays a vital role in its preference for pro-survival mechanisms in the presence of simvastatin. Such an effect of simvastatin has the potential to inhibit inflammation in neuronal cells of AD patients. This has been observed in humans and mouse models of AD [33]. In that study, simvastatin was shown to improve memory deficits in AD patients and mouse models of AD. 

Another possible explanation of the anti-inflammatory effect of simvastatin is its effect on the Ras family proteins [34]. The activation of Ras proteins, small GTPases, triggers the activation of mitogen-activated protein kinase (MAPK) pathways, which has been reported to activate NFκB downstream. Ras signaling contributes to the increase of oxidative stress and the induction of proinflammatory activities [35]. Inhibiting Ras prenylation via statin is one of the possible pleiotropic effects that has significant benefits in reducing oxidative damage and inflammation in AD [36]. ROS levels also control cellular signaling via NFκB signaling [37]. Again, the fate of signal transduction is dependent on the levels of ROS produced by cells. In AD, the accumulation of lipofuscin and ROS are common pathological hallmarks [38]. The use of simvastatin has the potential to reduce the inflammatory reaction of such ROS accumulation and redirect the neuronal machinery toward a pro-survival path. Apart from the above, simvastatin has been reported to reduce ROS levels in the tissues of rat models of heart diseases [39]. 

### 4.2. Reduction of Amyloid Beta and Tau Phosphorylation 

Amyloid beta accumulation and tauopathy are the two major biomarkers of AD. Simvastatin has been reported to lower both the accumulation of amyloid beta and tauopathy.

The first report on simvastatin’s effect in reducing amyloid beta was published in 2001 [40]. Those findings are based on a strong observed reduction of cerebral Aβ42 and Aβ40 in guinea pigs treated with high doses of the drug. Wolozin et al. (2007) then analyzed the data from the US Veterans Affairs database and reported simvastatin’s association with a strong reduction (by approximately more than 50%) in the incidence of Parkinson’s and other forms of dementia, including AD [28]. 

More recently, simvastatin was shown to reduce the levels of amyloid beta in yeast models of AD [41]. One of the explanations for this is its effect on Ras family proteins, as described above, along with its role in the processing of the amyloid precursor protein (APP) to the non-amyloidogenic pathway [42]. Perhaps most importantly, one of the enzymes that cleaves amyloid beta from its precursor protein, APP, is the enzyme BACE1. BACE1 is a prenylated protein, and its prenylation possibly plays a key role in its dimerization, as statins are found to reduce BACE1 dimerization [43]. Intriguingly, simvastatin also reduced the levels of amyloid beta in yeast models, where BACE prenylation plays no role in amyloid beta formation [41]. In the same study, the researchers also demonstrated that the inhibition of ergosterol synthesis by another antimicrobial which inhibits the pathway downstream had no effect in reducing amyloid beta levels. It is certainly interesting that the reduction of amyloid beta by simvastatin is not only the result of reducing ergosterol synthesis or the inhibition of BACE prenylation. Further investigations need to be conducted to understand the anti-amyloid role of simvastatin. Based on the origin of tau proteins, two different mechanisms have been proposed to explain the presence/accumulation of neurofibrillary tau tangles in AD. The first one suggests that the aggregation of the protein occurs from within the cell in an autonomous manner, while the second one states that tau assemblies cross from cell to cell, just like in prion infections. No matter what the process of the genesis of tau aggregates may be, simvastatin has the potential to lower the levels of protein aggregates, either by reducing cholesterol levels or by inducing protein clearance mechanisms. Recently, in an in vitro study, cholesterol dysregulation was found to be the key process involved in the entry of tau assemblies [44]. Hence, simvastatin’s ability to reduce cholesterol levels has a direct impact on reducing tauopathy. In a randomized controlled trial involving the treatment of hypercholesterolemic subjects with simvastatin or pravastatin, simvastatin but not pravastatin was found to lower the CSF levels of phospho-tau-181 [45]. Furthermore, in a separate study involving the mouse model of tauopathy, simvastatin markedly decreased the neurofibrillary tau tangles [46]. These findings suggest simvastatin’s effect on both the major biomarkers of AD. Although Ras signaling plays vital role in neuronal development and synaptic transmission, the inhibition of prenylation of the members of the Ras protein family has been found to reduce the accumulation of the tau protein, which is a key process in AD pathology [47].

### 4.3. Penetration of Blood Brain Barrier

One of the interesting facts about cholesterol transport is that cholesterol in general cannot cross the blood brain barrier (BBB) [48]. Hence, the pools of serum cholesterol and brain cholesterol remain separate. However, it is also known that oxidized cholesterol can cross the blood brain barrier, providing a theoretical explanation of how the two pools of cholesterol may affect each other by slight modifications in the chemical structure [49]. In AD patients, an increase in plasma hydroxycholesterol provides evidence of how cholesterol transports from brain to serum through the BBB [49]. Statins are designed to have their cholesterol lowering activity in the liver. Although cholesterol cannot cross the BBB by itself, statins can cross the BBB and have potential benefits in a range of brain disorders including AD. Among the statins, simvastatin is the most lipophilic and has the greatest ability to cross the blood brain barrier [50]. In a randomized controlled trial, simvastatin significantly increased the brain CSF phospholipid transfer protein and lowered apolipoprotein E levels; this can be attributed to its higher potential to cross the BBB [51]. This may also account for simvastatin being a powerful statin in terms of reducing the levels of amyloid beta in the brains of guinea pigs, neurofibrillary tau tangles in mouse models, and phospho-tau 181 in CSF in humans [40,45,46]. 

### 4.4. Mitochondrial Health

The observation of higher petite mutants in simvastatin (higher dosage)-treated yeast cells via the loss of mitochondrial DNA signifies the potential impact of simvastatin on mitochondrial function [9]. Another pleiotropic effect of the inhibition of HMGCR by simvastatin is its effect on cytochrome oxidases, which are crucial enzymes in mitochondrial function [52]. However, the statin-associated inhibition of HMGCR is partial, and its effect on cytochrome oxidases is also partial. In a previous study, it was reported that amyloid beta interacts with the cytochrome c oxidases and causes mitochondrial dysfunction [53]. Considering the partial reduction of cytochrome c oxidase activity and the strong reduction of amyloid beta in AD, it is beneficial for mitochondrial health. Simvastatin usage could potentially reduce the interacting cytochrome oxidases and prevent amyloid beta-associated mitochondrial impairment, restoring its function. Durhuus et al. reported that the long-term treatment of hypercholesterolemic patients with simvastatin improved mitochondrial respiration in peripheral blood mononuclear cells and platelets, suggesting its positive impact on mitochondrial health [54]. On the other hand, simvastatin has also been reported to enhance mitochondrial turnover via enhancing mitophagy [39]. During AD, the impairment of mitochondrial turnover and mitochondrial dysfunction are commonly observed in neuronal cells. Simvastatin usage in such conditions could therefore have beneficial effects on mitochondrial health. 

### 4.5. Improvement of Blood Flow 

The widely accepted explanation for the inadequate blood supply and the resultant hypoxia observed in AD is endothelial dysfunction. Furthermore, in a study involving mice, the hypoxic condition was found to contribute toward higher accumulation of amyloid beta in exosomes via the regulation of γ-secretases [55]. Interestingly, amyloid beta was reported to have detrimental effects on the peripheral as well as cerebral endothelium in an in vitro study [56]. All these studies correlate well together and signify the role of amyloid beta in causing hypoxia, and, in turn, of hypoxia causing the accumulation of amyloid beta. Both these events contribute to the degradation of vascular health. However, it is not yet clear which event occurs first.

The known effect of simvastatin is the lowering of cholesterol levels and the reduction of atherosclerotic plaques in the blood vessels, both of which have significant effects on the vascular health of AD patients. Among the statins, simvastatin was found to have the highest activity in terms of increasing the expression of NOS3, a hallmark of a functional endothelium, in induced pluripotent stem cells (iPSCs) differentiated as endothelial cells. There were around 1019 upregulated genes and 1600 downregulated genes in these cells. Genes involved in processes such as the anti-inflammatory response, angiogenesis, and extracellular matrix organization were found to be upregulated, all of which contribute to a functional endothelium. In summary, simvastatin was shown to improve endothelial cell function in both diabetic and non-diabetic conditions by inhibiting geranylgeranyl transferase I activity [57]. Based on these findings, it can be said that simvastatin can improve vascular health and improve blood flow in AD patients. 

### 4.6. Proteostasis

The loss of proteostasis is a common pathological feature of AD, of which the accumulation of amyloid plaques and tauopathies are the consequences [58]. Several aspects of protein homeostasis are disrupted and damaged irreversibly during AD progression, which ultimately leads to the development of the disease. Proteostasis is connected to several eukaryotic fundamental processes that include the immune system, energy metabolism, vesicle mediated trafficking, the recycling of the cellular materials, synaptic functions, lipid metabolism, stress response, and others. 

**Figure 3:**
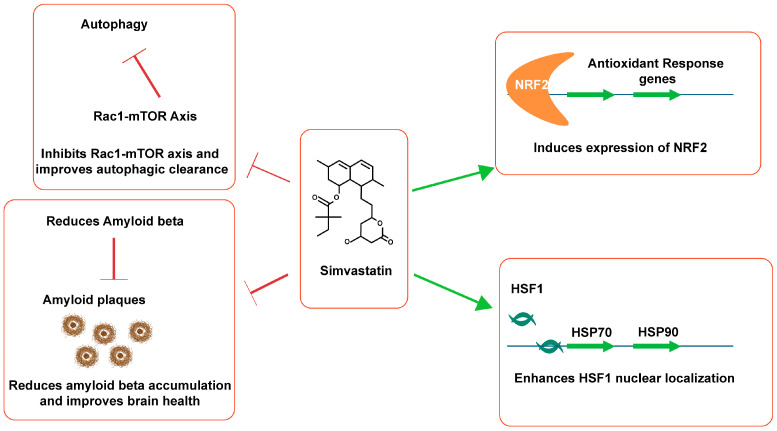
The effects of simvastatin on the overall proteostasis that relates to the AD pathology. The pointed arrows are used to show the increase in the expression of the genes and blunt arrows are used to show the inhibition of the target mechanism. NRF2, Nuclear factor erythroid 2-related factor 2; mTOR, mammalian target of rapamycin; HSF1, heat shock factor 1; and HSP, heat shock protein.

Simvastatin has been reported to have a multifactorial effect on several of these pathways, but the one that is of high importance is its effect on cellular protein quality control (refer to Figure 3). As early as 2006, simvastatin was reported to act on heat shock factor 1 in endothelial cells and improve their vascular efficiency [59]. In that study, simvastatin was shown to increase the expression of heat shock protein (HSP) 70 and HSP90. Specifically, the expression of HSP70 is associated with the cell survival pathway, while HSP90 may play roles in apoptotic inductions. In a more recent study, simvastatin was found to inhibit HSP90 in triple negative breast cancer cells [60]. These findings suggest simvastatin’s significant impact on the heat shock response. The heat shock response is the first line of cellular protein quality control. 

Simvastatin was also found to enhance antioxidant activity via nuclear factor erythroid 2-related factor 2 (NRF2) and reduce endoplasmic reticulum stress in hepatic cells [61]. Such activity of simvastatin could be beneficial in AD pathology, as chronic ER stress is a common feature of AD. Furthermore, simvastatin inhibits the Rac1-mTOR pathway in coronary arterial myocytes (CAMs), thereby increasing the numbers of autophagosomes and enhancing autophagosome lysosome fusion [62]. During AD, the accumulation of autophagosomes and impairment of their clearance are major events as the disease progresses. If simvastatin acts in neurons in similar manner to in CAMs, neuronal health is likely to improve due to simvastatin’s potential to reduce the aggregation of unwanted proteins. Perhaps, simvastatin’s abilities to strongly reduce amyloid beta and lower tauopathy can be attributed to this mechanism [40,46]. Simvastatin not only improved autophagic clearance in CAMs, but it also enhanced the lysosomal biogenesis [62]. 

As mentioned previously, yeast cells and human cells share certain biological characteristics. For example, yeast has considerable utility as a model for aging [63]. Aging yeast and aging human cells show reduced proteostasis, which means that some deleterious proteins accumulate with age. The accumulation of amyloid beta in yeast can be readily observed by comparing a population of aged yeast cells with a young population; aged cells are larger and can be separated using a cell sorter [64]. In addition, amyloid beta can be visualized by fusing it to fluorescent proteins such as GFP, where it can be observed in cells by fluorescence microscopy [65]. Examinations of cell populations show that young cells remove amyloid beta fused to GFP, while in older (larger) cells, the amyloid beta fused to GFP persists [63]. When the yeast is grown in the presence of simvastatin, the levels of amyloid beta or amyloid beta fused to GFP are about 10% of those in untreated cells [41]. A possible explanation for this is that simvastatin causes increased proteostasis; however, other explanations should be considered. 

### 4.7. Controversies and Clinical Trials

Many have dismissed statins as having little to no effect on AD; however, there are several points to consider when addressing what clinical studies have suggested so far.

First, data from numerous epidemiology studies have tried to address the issue of whether statins can be used to treat AD, as it is typically diagnosed years after the actual onset of the disease. It is doubtful whether any drugs can reverse the effects of AD, as the majority of the changes that occur are irreversible as AD progresses; however, things can be done to prevent AD or to slow its progression. 

Second, it is clear from many studies that not all statins are the same in terms of their effects on AD, and studies should examine statins individually. We argue that when simvastatin data are not separated from data on other statins which have little impact on AD, the data often show no or reduced significance. Therefore, the most relevant studies are those that involve the prevention of AD. The study by Wolozin et al. (2007) is especially significant, because it examined a younger cohort ( > 65 yo) of people from the Veteran’s Affairs administration who were prescribed a statin to treat hypercholesterolemia [28]. The study separated subjects into groups according to which statin they were prescribed and whether they were diagnosed with dementia after 3 years. The group taking simvastatin had a 50% reduction in the incidence of dementia, while those taking atorvastatin showed only a very moderate reduction. We are not aware of any other study that has looked at the effects of statins in this way; however other clinical trials and relevant studies are listed in Table 2.

## 5. Conclusions

Simvastatin is a modified product of *Aspergillus terreus* that has a well-established role as a life-saving drug, i.e., in the treatment of hypercholesterolemia. In addition to this, beneficial side effects, including antimicrobial activity and the prevention of AD, have been discussed in this review. Some of the antimicrobial effects of simvastatin can be explained by its inhibition of HMGCR; however, some effects appear to be due to its inhibition of novel targets. Likewise, some of simvastatin’s effects appear to be due to its inhibition of novel targets. 

We hope that this discussion of the literature relating to the beneficial effects of simvastatin may serve to encourage further investigation and exploitation of the compound. 

## Figures and Tables

**Table 1:** Antimicrobial effects of simvastatin.

**Microorganism**	**Effect**	**Mechanism of action**	**Concentration**	**Reference**
*Candida albicans*	Growth inhibition	ND	100 µM	[8]
*Aspergillus fumigatus*	Growth inhibition	ND	0.1 µM	[8]
*Candida glabrata*	Growth inhibition	Reduce ergosterol. Causes loss of mitochondrial DNA.	20 µM	[9]
*Aspergillus* spp.	Growth inhibition	ND	MIC ranging from 16 to 108 µg/mL except for *A. niger* and *A. terreus* which were >256 µg/mL. MFC ranging from 32–128 µg/mL for those that are determined.	[10]
*Escherichia coli*	Growth inhibition	Inhibits chaperone protein DnaK (a homologue of mammalian HSP70) Inactivation of AcrAB-TolC efflux pump. Alters amino acid synthesis pathway, ribosomal protein synthesis, TCA cycle and glyoxylate dicarboxylate metabolism.	MIC: 128 µg/mL	[11]
Methicillin sensitive *Staphylococcus aureus* (MSSA) ATTC 25213	Growth inhibition	Inhibits macromolecular synthesis pathway (DNA replication, RNA synthesis, protein synthesis, lipid synthesis, cell wall synthesis) Enhances protein degradation. Inhibits *S. aureus* toxin production. Reduces biofilm formation. Reduces inflammatory cytokines induced by bacterial infection in skin. Synergistic activity with other antimicrobials.	MIC: 41.67 ± 18.04 µg/mL	[12,13,14]
Methicillin resistant *Staphylococcus aureus* SA (MRSA) ATTC 43300	Growth inhibition		MIC: 83.33 ± 36.08 µg/mL	
Vancomycin sensitive *Enterococcus* ATTC 19433	Growth inhibition	ND	MIC: 83.33 ± 36.08 µg/mL	
*Acinetobacter baumannii* ATTC 17978	Growth inhibition	ND	MIC: 15.62 ± 0.00 µg/mL	
*Enterrobacter aerogenes* ATTC 29751	Growth inhibition	ND	MIC: 15.62 ± 0.00 µg/mL	
*Trichophyton rubrum* ATCC 28188	Growth inhibition	ND	MIC: 6.25–12.5 µg/mL	[15]
*Trichophyton mentagrophytes* ATCC 9533	Growth inhibition	ND	MIC: 6.25 µg/mL	
*Microsporum gypseum* (ATCC 24102)	Growth inhibition	ND	MIC: 6.25–12.5 µg/mL	
*Microsporum canis* (ATCC 36299)	Growth inhibition	ND	MIC: 6.25–12.5 µg/mL	
*Aggregatibacter actinomycetemcomitans*	Growth inhibition	ND	MIC: <1 µg/mL	[16,17]
*Porphyromonas gingivalis*	Growth inhibition	ND	MIC: 2 µg/mL	
*Mycobacterium ulcerans*	Growth inhibition (1.7-fold reduction in colony forming unit (CFU))	ND	IC50: 54.13 μg/mL	[18]

Footnotes. MIC, Minimum Inhibitory Concentration; MFC, Minimum fungicidal concentration; ND, not defined; and IC50, Inhibitory Concentration with 50% growth inhibition.

**Table 2:** Insights into simvastatin usage in AD from clinical trials and some relevant studies.

Year	Study Design	Findings	Ref	Remarks
**2002**	Randomized, placebo-controlled, double-blind study. Research Question: Do statins alter cholesterol metabolites and reduce amyloid beta (Aβ) levels in the cerebrospinal fluid of patients with Alzheimer’s disease? Participants: 44 patients	Simvastatin significantly decreased Aβ40 and 24S-hydroxycholesterol levels in the cerebrospinal fluid of patients with mild AD. These changes were not observed in more severely affected patients.	[66]	The findings suggest benefits of using simvastatin in mild AD, but not in severe AD.
**2003**	Clinical and biological effects of cholesterol-lowering treatment with a statin Participants: 19 patients with Alzheimer’s disease. Statin Dosage: Simvastatin 20 mg/day for 12 weeks. Outcome measurement: Changes after 12 weeks in the cerebrospinal fluid (CSF) levels of, Aβ42, α-secretase-cleaved amyloid precursor protein (α-sAPP), β-secretase-cleaved APP (β-sAPP), tau, phospho-tau and the plasma levels of Aβ42. Alzheimer’s Disease Assessment Scale-Cognition (ADAS-cog) score.	CSF α-sAPP and CSF β-sAPP were significantly reduced, but the CSF levels of tau, phospho-tau, Aβ42, and the plasma levels of Aβ42 were unchanged. The ADAS-cog score was slightly increased (*p* < 0.05). Simvastatin acts directly on the processing of APP by inhibiting both the α- and the β-secretase pathways.	[67]	Simvastatin reduced APP processing indicating potential in reducing Aβ42 if administered early in life. However, patients of AD may not benefit much as the irreversible damage to the brain persists in AD patients.
**2007**	Data from the decision support system of the US Veterans Affairs database, which contain diagnostic, medication, and demographic information on 4.5 million subjects. Participants: 700,000 subjects taking simvastatin for hypercholesterolemia and over 50,000 subjects taking atorvastatin who were aged >64 years.	Simvastatin strongly reduced the incidence of dementia, and atorvastatin showed slight reduction.	[28]	Perhaps this is the only prospective study with data from sufficient subjects at an early age group of ( ≥ 65 years). Simvastatin has a strong potential as preventative by reducing the incidence of AD.
**2007**	Cognitively normal subjects: Brain autopsies on 110 subjects, aged 65 to 79 years. Statin Usage: Simvastatin, pravastatin, lovastatin, or atorvastatin user vs nonuser.	The risk for typical AD pathology was reduced in statin users and there was an association between antecedent statin use and neurofibrillary tangle burden at autopsy.	[68]	Simvastatin reduces neurofibrillary tau tangles.
**2011**	Randomized, double-blind, placebo-controlled trial. Participants: Mild to moderate AD and normal lipid levels. Statin usage: Simvastatin, 20 mg/day, for 6 weeks then 40 mg per day for the remainder of 18 months or identical placebo. Outcome measurement: Alzheimer’s Disease Assessment Scale-cognitive portion (ADAS-Cog) and clinical global change, cognition, function, and behaviour.	Simvastatin lowered lipid levels but had no effect on change in ADAS-Cog score or the secondary outcome measures. Simvastatin had no benefit on the progression of symptoms in individuals with mild to moderate AD despite significant lowering of cholesterol.	[69]	Simvastatin may not be suitable for treatment of mild and moderate AD.
**2016**	Double-blind, randomised, placebo-controlled trials. Statin dosage: Administered for at least 12 months to people at risk of dementia. Participants: All participants had a history of, or risk factors for, vascular disease: 26,340 participants aged 40 to 82 years of whom 11,610 were aged 70 or older.	Statins (pravastatin and simvastatin) given late in life did not prevent dementia.	[70]	Simvastatin may not be beneficial for prospective AD patients after short term usage.
**2017**	Analysis of datasets of integrated clinical trials, and prospective observational studies.	The incidence of AD was significantly lower in statin users. ApoE4/ApoE4 AD patients treated with statins showed better cognitive function over the course of 10-year follow-up.	[71]	Long term studies may help understand the benefits of statins in AD patients.
**2023**	A longitudinal cohort study using the Swedish Registry for Cognitive/Dementia Disorders: 15,586 patients with mean age of 79.5 years at diagnosis and mostly women (59.2 %).	Taking one defined daily dose of statins on average was associated with more MMSE points after 3 years compared to no use of statins. Simvastatin users showed more MMSE points after 3 years compared to atorvastatin and rosuvastatin users. Younger simvastatin users had more MMSE points compared to younger atorvastatin users after 3 years.	[72]	Simvastatin performed better than other statins; younger simvastatin users benefited more.
